# Potential biomarkers for neuroinflammation and neurodegeneration at short and long term after neonatal hypoxic-ischemic insult in rat

**DOI:** 10.1186/s12974-019-1595-0

**Published:** 2019-10-28

**Authors:** Nozha Borjini, Sandra Sivilia, Alessandro Giuliani, Mercedes Fernandez, Luciana Giardino, Fabrizio Facchinetti, Laura Calzà

**Affiliations:** 1 Corporate Pre-clinical R&D, Chiesi Farmaceutici S.p.A, Largo Belloli 11/A, 43122 Parma, Italy; 2 Health Science and Technologies Interdepartmental Center for Industrial Research, University of Bologna, Via Tolara di Sopra 41/E, I-40064 Ozzano Emilia, BO Italy; 3 IRET Foundation, Via Tolara di Sopra 41/E, 40064 Ozzano Emilia, BO Italy; 4 Department of Veterinary Medical Sciences, University of Bologna, Via Tolara di Sopra 50, 40064 Ozzano Emilia, BO Italy; 5 Department of Pharmacy and Biotechnology, University of Bologna, Via Tolara di Sopra 41, 40064 Ozzano Emilia, BO Italy

**Keywords:** Neonatal hypoxia-ischemia, Inflammatory biomarkers, Neurological disorders

## Abstract

**Background:**

Hypoxic-ischemic (HI) encephalopathy causes life-long morbidity and premature mortality in term neonates. Therapies in addition to whole-body cooling are under development to treat the neonate at risk for HI encephalopathy, but are not a quickly measured serum inflammatory or neuronal biomarkers to rapidly and accurately identify brain injury in order to follow the efficacy of therapies.

**Methods:**

In order to identify potential biomarkers for early inflammatory and neurodegenerative events after neonatal hypoxia-ischemia, both male and female Wistar rat pups at postnatal day 7 (P7) were used and had their right carotid artery permanently doubly occluded and exposed to 8% oxygen for 90 min. Sensory and cognitive parameters were assessed by open field, rotarod, CatWalk, and Morris water maze (MWM) test. Plasma and CSF biomarkers were investigated on the acute (24 h and 72 h) and chronic phase (4 weeks). Brains were assessed for gene expression analysis by quantitative RT-PCR Array.

**Results:**

We found a delay of neurological reflex maturation in HI rats. We observed anxiolytic-like baseline behavior in males more than females following HI injury. HI rats held on the rotarod for a shorter time comparing to sham. HI injury impaired spatial learning ability on MWM test. The CatWalk assessment demonstrated a long-term deficit in gait parameters related to the hind paw. Proinflammatory biomarkers such as IL-6 in plasma and CCL2 and TNF-α in CSF showed an upregulation at 24 h after HI while other cytokines, such as IL-17A and CCL5, were upregulated after 72 h in CSF. At 24 h post-injury, we observed an increase of Edn1, Hif1-α, and Mmp9 mRNA levels in the ipsilateral vs the contralateral hemisphere of HI rats. An upregulation of genes involved with clotting and hematopoietic processes was observed 72 h post-injury.

**Conclusions:**

Our work showed that, in the immature brain, the HI injury induced an early increased production of several proinflammatory mediators detectable in plasma and CSF, followed by tissue damage in the hypoxic hemisphere and short-term as well as long-lasting neurobehavioral deficits.

## Background

Despite the advances in perinatal care, hypoxic-ischemic encephalopathy remains a major clinical problem. Hypoxia-ischemia (HI) is a contributing factor to neonatal morbidity and mortality, often leading to chronic neurological disorders and disabilities, such as mental retardation, motor and behavioral developmental problems, cerebral palsy, seizure, and epilepsy [1–4]. The introduction of therapeutic hypothermia, while beneficial, still leaves many treated infants with lifelong behavioral, social, attentional, cognitive, and functional motor deficits [5, 6].

The susceptibility of the immature central nervous system (CNS) to HI is largely dependent on the temporal and regional status of critical developmental processes, as well as on the regulation of cerebral blood flow and metabolism [7]. The clinical diagnosis of neonatal HI and the assessment of disease severity mainly relies on the Sarnat score, brain CT (computed tomography) scans [8], MRI (magnetic resonance imaging), ultrasound diagnosis, and EEG (electroencephalogram) detection methods [9, 10]. Because of the influence of the progressive disease process and other factors, the Sarnat score is subjective, and other tests have limitations and double effectiveness in premature/newborn infants. For example, after neonatal HI onset, there is a time range of 24 h between metabolism changes and pathological alterations leading to morphological changes in the brain. The appearance of CNS damage using neuroimaging techniques can take up to 72 h [11, 12]. Thus, the early clinical detection of blood or CSF biomarkers might have a prognostic value, also allowing a treatment monitoring, compared with MRI or CT results.

Post-ischemic neuroinflammation in the immature brain is a key pathophysiological factor in the development of HI-related injury [13–16]. However, the course of the inflammatory process has been investigated only partly in the neonatal setting [17, 18]. Several studies have shown that neonatal HI triggers’ extensive inflammatory reactions in the brain which includes activation of the innate immune system [19] and experimental studies in neonatal animals have demonstrated that inhibition of proinflammatory biomediators is neuroprotective [19–21]. The early neuroinflammatory response is associated with the production of several immune active cytokines/chemokines, microglia activation, and infiltration of peripheral immune cells [1, 10, 22, 23], which aggravates the brain injury outcomes. However, this early response that initially is harmful could be beneficial in the later stage by contributing to the restoration of tissue homeostasis [17, 24].

The identification of early noninvasive biomarkers of disease is a vital question, especially during the first period of a lifetime, since it could provide valuable, beneficial, and advanced diagnostic evidence when clinical and radiological signs are still silent. Therefore, this study aimed to identify potential biomarkers for the mechanisms underlying hypoxic-ischemic injury and the subsequent neuroinflammatory response in a validated preclinical model of HI, focusing on the levels of inflammatory mediators in plasma and CSF, and compare their levels with the neurological disorders. We observed that cerebral hypoxic ischemia induces an early neuroinflammatory response likely contributing to delayed cellular death that extends over several days, eventually leading to the extensive brain hemisphere atrophy. Here we demonstrate that, despite the nervous system’s attempt to recover in the acute phase by upregulating blood clotting and hematopoietic genes, the HI insult leads to short-term as well as long-lasting behavioral deficits in rats up to 4 weeks after the injury. Sex differences in the response to cerebral HI have also been examined in this study.

## Methods

### Animals and experimental groups

A total of 65 Wistar rat pups at postnatal day 7 (P7) of both sexes were used in this study. Rats were weaned at the age of 21 days. Animals were maintained in an animal room on a 12-h light/12-h dark cycle and at constant temperature (22 ± 2 °C), food and water ad libitum. All animal protocols described herein were carried out in accordance with the European Community Council Directives (86/609/EEC), approved by the intramural ethical committee for animal experimentation of Bologna University and complied with the guidelines published in the *NIH Guide for the Care and Use of Laboratory.* The animals were divided into three experimental groups: (a) 24 h HI (*n* = 7), 24 h sham (*n* = 6); (b) 72 h HI (*n* = 9), 72 h sham (*n* = 6); (c) P44 HI male (*n* = 13), P44 sham male (*n* = 8), P44 HI female (*n* = 6), P44 sham female (*n* = 7) (*P*, postnatal day).

### Inclusion and exclusion criteria

Rat pups were randomized among litters and pups from the same litter were served for both HI and sham groups. Pups with weight less than 12 g and higher than 14 g were excluded from the experiment. As in a pilot study performed prior to the actual experiments, most of the rats with weight less than 12 g did not survive the hypoxia-ischemia procedure, while pups with weight more than 14 g had a variable outcome.

### Neonatal hypoxia-ischemia injury model

An accepted rodent model of neonatal asphyxia is a modification of the Levine [25] done by Rice et al., [26], consisting in the combination of ischemia, achieved by unilateral occlusion of carotid artery, followed by exposure to hypoxia in 7-day-old rats. Indeed in rats, hypoxic seizures could be induced during the critical developmental window, P6–12, which is a period of synaptic maturation and corresponds to the age dependence of clinical hypoxia-associated neonatal seizures [27–30] and is thought to match with a human premature brain of 32–36 weeks of gestation. The most widely used animal model is the unilateral common carotid artery ligation followed by exposure to hypoxia in rats at P7. The surgery was performed on Wistar rats at P7 under a surgical microscope as described previously [26] with an introduction of some modifications. In brief, the pup was first weighed and then anesthetized with 3% isoflurane. The surgery lasted less than 5 min. After placing the rat on a surgical heating pad at 37 °C, the skin was cleaned with 10% povidone iodide and a less than 1-cm longitudinal midline incision of the neck was performed to expose the right common carotid artery (CCA). The fibrous sheath that wraps together both the carotid and the vagus nerve was broken and separated in order to avoid an overextension of the nerve. The CCA was permanently doubly ligated with a 5/0 silk suture. After the ligation, few drops of surgical glue were used for the suture of the skin. Pups were placed above a heat mat at 37 °C until awakening and recovering, then were returned to their dam, and were allowed to recuperate for 1.5 h. Pups were then placed in a hypoxic chamber that contained 8% O^2^ and 92% N^2^ with a constant flow of 3 L/min for 90 min, submerged in a water bath maintained at 32 °C, which is the usual temperature to which rat pups are exposed when huddling with their mother [31, 32]. After hypoxia, all pups were returned again to their dam for recovery. Sham animals underwent the HI surgical procedures (i.e., exposure of the CCA) without artery ligation and without exposure to hypoxic conditions.

### Short-term neurofunctional outcome following cerebral HI

The examination of neurobehavioral development was performed for all rat pups from P8 to P21 after the hypoxic-ischemic insult and was carried out daily between 10 and 12 a.m. (Additional file 1: Figure S1). Body weights of rat pups were recorded daily. Pups were tested for the following neurological reflexes: (1) *Righting reflex*: this test is believed to be a reflection of subcortical maturation estimate, the generation of these movements from circuits in the spine connected to the supplementary motor area, the basal ganglia, and the reticular formation. The time (seconds) used by the animal to go from a supine to a prone position by placing all four paws on the surface was recorded. (2) *Negative geotaxis*: this test examines the sensorimotor function of neonatal rats [33]. Rat pups were placed upside down in the middle of a slope (45°) of 30 cm. The latency to turn 180° to an upward direction was recorded. From the day when the animal turns to go up, the time (seconds) it took to reach the upper side of the plane was recorded. The maximum duration of the recording was 30 s; otherwise, the test was considered negative. (3) *Sensory reflex*: the ear and the eyelid of the pup were touched with a cotton swab and the first day of the ear twitch reflex and the contraction of the eyelid were recorded. (4) *Auditory startle*: the first day of the startle response to a clapping sound was observed. (5) *Crossed extensor reflex*: the left hind paw was pinched and the possible extension of the right paw was recorded. (6) *Limb placing*: the back of the forepaw and hind paw was touched with the edge of the bench while the animal is suspended, and the first day of lifting and placing the paws on the table was noted. (7) *Limb grasp*: the forelimbs were touched with a thin rod, and the first day of grasping onto the rod was recorded. (8) *Gait*: the animals are placed at the center of a white plexiglass circle (Ø = 13 cm). Register the day when they start to move out of the circle with both front paws, estimate the time (seconds) that the animal uses to exit out of the circle. In the case in which the animal does not leave the circle within 30 s, the test is considered negative. In order to assess the development of neurological reflexes, rats are given a score to the corresponding time (seconds). The higher score indicates greater capacity for the development of neurological reflexes.

### Long-term neurofunctional outcome after HI insult

The assessment of long-term neurofunctional handicap was performed in sham and HI groups 3 weeks after the insult (P28). These tasks consisted of the open-field, rotarod, Catwalk, and Morris water maze (MWM) behavior tests (Additional file 1: Figure S1).

#### Open field

Animals were observed for locomotor behavior in an open field. Pups were placed in an open field consisting of a 46 × 46-cm plastic chamber with 41-cm high walls around, with a dark gray floor virtually divided into 16 fields by the software. Rats were placed individually in the center of the chamber always facing the same direction, and the latency to leave this first square was recorded. The following parameters were measured using AnyMaze Video tracking software (AnyMaze, Stoelting, Wood Dale, IL, USA): distance traveled, rearing, grooming, and ambulation frequency. Speed was calculated from the ambulatory time and the total traveled distance. Animals were video-recorded for 10 min [34].

#### Rotarod

The rotarod test (LE 8500 RotaRod: 2Biological Instruments, Varese, Italy) consists on a 2-day test. Animals were exposed to one habituation session during 3 min in the apparatus on slow velocity (20 rpm). In the test session, 24 h later, the animal’s motor ability was evaluated. The rotarod test was performed by placing rats on rotating drums and measuring the time each animal maintained its balance on the rod. The acceleration rate of the rotarod was from 16 to 40 rpm over a 6-min period. The variables recorded were the latency of the first downfall, the number of falls (maximum of three), and the time of permanence in the apparatus [35, 36].

#### CatWalk

Cortical function was analyzed by CatWalk (Noldus Information Technology, Wageningen, The Netherlands), a quantitative gait analysis system. Each rat ran across a glass walkway transversely, three complete runs were recorded using a camera positioned below, and the average was calculated. If an animal failed to complete a run within 5 s, walked backwards, or reared during the run, the process was repeated. The experiment was performed in the dark; the glass walkway was illuminated with beams of light, thereby allowing the animals’ paws to reflect light as they touched the glass floor. Each paw was labeled on the recorded video in order to calculate paw-related parameters. The gait-related parameters measured using the CatWalk system were the following: *maximum contact area*: the maximum area of a paw that comes into contact with the glass plate; *stand*: stance phase is the duration in seconds of contact of a paw with the glass plate and swing speed is the speed (distance unit/second) of the paw during swing. The formula of *swing speed* is swing (seconds) phase which is the duration in seconds of no contact of a paw with the glass plate. For the stride length which is the distance (in distance units) between successive placements of the same paw, the calculation of stride length is based on the X-coordinates of the center of the paw print of two consecutive placements of the same paw during max contact and taking into account Pythagoras’ theorem [37].

#### Morris water maze

Three weeks after HI lesion, the spatial memory performance was evaluated using an MWM (180 cm diameter, 45 cm high) virtually divided in four equal imaginary quadrants by the AnyMaze software. The water of the pool was made opaque by using non-toxic gray tempera paint. The water level was 1 cm above the platform made of transparent plexiglass (10 cm diameter). The training consisted of a swim followed by a 30 s platform sit. The escape latency to find the platform was measured for individual animals on each day. The experimenter guided rats that did not find the platform within 120 s to it. To assess long-term memory, 24 h after the final trial, the platform was removed from the maze and a 2-min free swim will be conducted, and the time (seconds) spent during the first 20 s and the entire swim in the quadrant formerly occupied by the platform will be recorded [38].

### CSF and plasma biomarker analysis

The method of CSF sampling was adapted from the method of Liu et al., [39, 40]. Briefly, the rat pup was anesthetized by isoflurane inhalation (isofluorane 4%) (Gas Anesthesia System-21100, Ugo Basile, Varese, Italy) and fixed by one investigator with the head positioned at a 90° angle. A sagittal incision of the skin was made below the occiput, and the subcutaneous tissue and neck muscles through the midline were separated and held apart using a microretractor. The dura mater of the cisterna magna was then penetrated by an 8-cm-long glass capillary, which had a narrowed tip with an inner diameter of about 0.3 mm so that the CSF flowed into the capillary. After collection, each sample was centrifuged at 2000×*g* for 10 min at 4 °C, and the supernatant was aliquoted and stored at − 80 °C for biochemical assays. Blood was collected from the abdominal aorta in EDTA-K2 Vacutainer tubes and centrifuged at 3000×*g* for 10 min at 4 °C, and the plasma was collected, aliquoted, and stored at − 80 °C until used.

Proteins known to play key roles in neuroinflammation pathways were selected. For this purpose, Bio-Plex Pro™ Rat Cytokine 24-plex Assay (Bio-Rad; Milano, Italy) was used. The kit included EPO, G-CSF (CSF3), GM-CSF (CSF2), GRO/KC, IFN-γ, IL-1α, IL-1β, IL-2, IL-4, IL-5, IL-6, IL-7, IL-10, IL-12p70, IL-13, IL-17A, IL-18, M-CSF (CSF1), MCP-1 (CCL2), MIP-1α (CCL3), MIP-3α (CCL20), RANTES (CCL5), TNF-α, and VEGF. The simultaneous quantification of the different proteins in CSF and plasma was performed using xMAP technology and a MAGPIX Luminex platform. This technology makes use of different populations of color-coded beads conjugated with monoclonal antibodies specific to a particular protein, thus allowing simultaneous capture and detection of specific analytes from a sample. All the beads from each set are read off, which further validates the results. Using this process, xMAP Technology allows multiplexing of up to 50 unique bioassays within a single sample, both rapidly and precisely [41, 42]. In brief, after the incubation of a specific monoclonal antibody conjugated bead population with 50 μl of CSF/plasma samples for 1 h at RT, washed beads were incubated with a detection antibody solution at RT for 30 min, then with the streptavidin–phycoerythrin conjugated solution (RT, 10 min). After washing, beads were resuspended in the assay buffer, shaken for 1 min, and then read on the MAGPIX instrument. The results were analyzed with xPONENT 4.2® software and expressed as picograms per milliliter.

### Gene expression analysis by quantitative RT-PCR Array

The total RNA was prepared from the brain using QIAzol Reagent, cleaned with RNeasy Mini kit (Qiagen; Milano- Italy), and eluted in RNase free water. The purity and concentration of RNA were evaluated by spectrophotometry using NanoDrop ND-2000 (ThermoScientific, Milano, Italy). Complementary DNA (cDNA) synthesis was performed using RT^2^ First Strand kit (Qiagen) following the manufacturer’s instructions. The expression analysis of 84 genes associated with hypoxia was carried out with the RT^2^ Profiler™ PCR Array Rat Hypoxia Signaling Pathway (PARN-032Z, Qiagen) according to the manufacturer’s guidelines in 96-well plates with a CFX96 Touch™ Real-Time PCR Detection System (Bio-Rad). The raw data obtained was uploaded into GeneGlobe for analysis. Relative quantification of messenger RNA (mRNA) expression was calculated using the comparative cycle threshold (CT) method and expressed as log2-fold change of expression. The fold change (2^−∆∆Ct^) is the normalized gene expression (2^−∆Ct^) in the test sample which is the ipsilateral hemisphere divided by the normalized gene expression (2^−∆Ct^) in the control sample (the contralateral hemisphere).

### Statistical analysis

Results in the appearance of physical and neurological reflexes as well as body weights were compared with Student’s *t* test. Statistical differences between groups for each outcome measured were analyzed using one-way ANOVA or two-way ANOVA followed by Tukey’s post hoc. All the data were expressed as mean ± SEM and significance was set at *P* ≤ 0.05. All statistical analyses were performed using GraphPad Prism 7.0 (GraphPad Software). For the normalization of gene expression on the RT^2^ PCR Profiler Array, data were normalized to five housekeeping genes included in the kit. The CT was determined for each sample and normalized to the average CT of the five housekeeping genes. A comparative CT method was used to calculate relative gene expression. Data are represented as log2-fold change relative to control. The *P* values were calculated on the basis of a Student’s *t* test of the replicate 2^−∆Ct^ values for each gene in the control group and treatment groups, and *P* values less than 0.05 were considered significant.

## Results

**Fig. 1:**
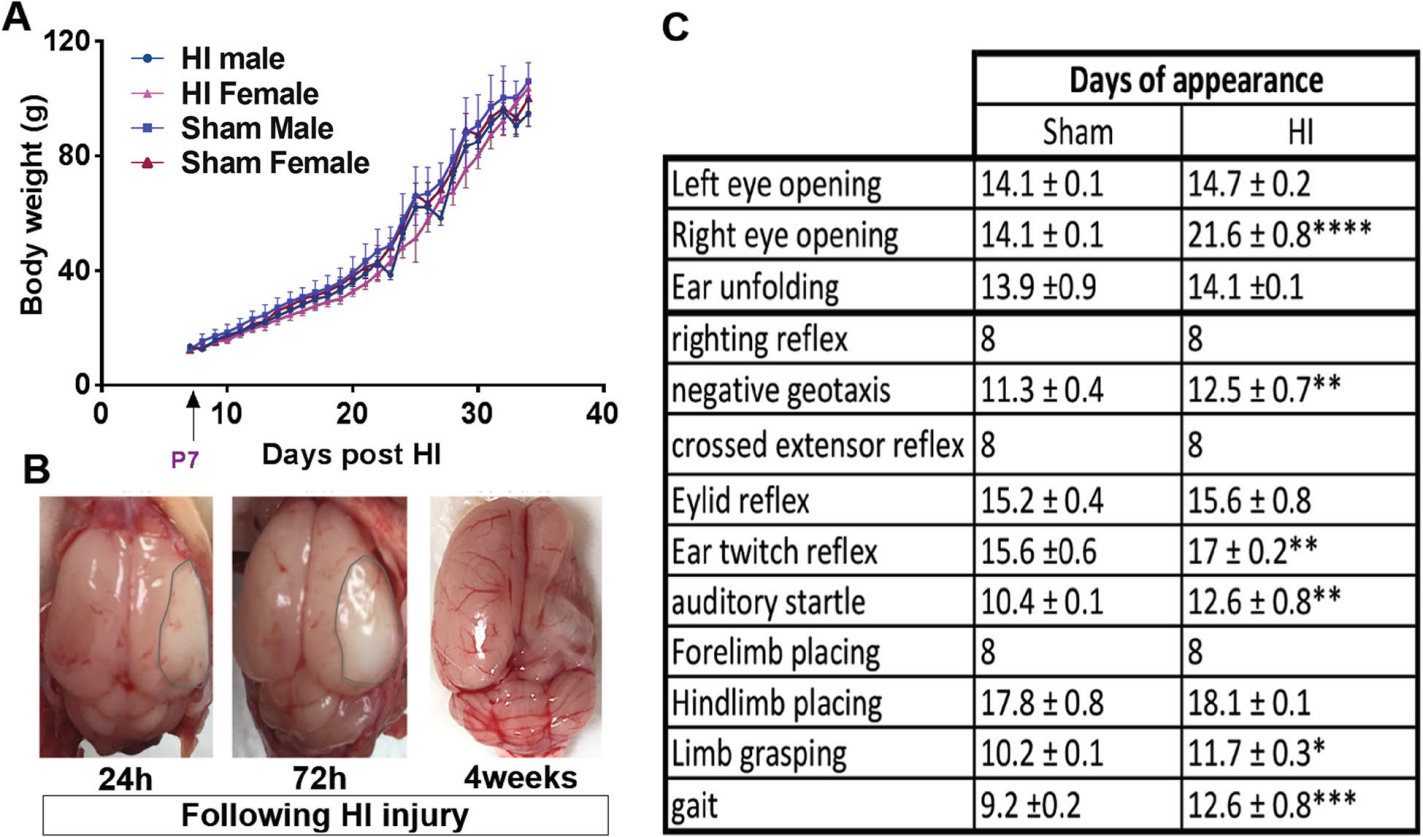
Hypoxia-ischemia induces tissue loss and delay of neurological reflexes. **a** Average weights ± S.E.M. of hypoxic and sham rats starting from P7 (1-day prior the intervention) (HI male (*n* = 13), sham male (*n* = 8), HI female (*n* = 6), sham female (*n* = 7)). **b** Representative images of brain lesion induced by acute (24 and 72 h) and chronic hypoxia-ischemia (4 weeks). **c** Average day ± S.E.M. of the appearance of physical and neurological reflexes in sham and HI rats. HI mice (*n* = 19), sham mice (*n* = 15). *P < 0.05, **P < 0.01, ***P < 0.001, ****P < 0.0001 vs. control rats

Three rats died during different steps of the experiment (mortality rate, 4.6%). Animals were weighed every day of life, and all along the experiment, there were no significant differences in body weight, neither between sham and HI groups nor between HI males and females (Fig. 1a). HI induced brain edema in the acute phase 24 and 72 h after unilateral ligation of the right carotid artery and hypoxia for 90 min in neonatal rat and tissue loss in the chronic phase, i.e., after 4 weeks (Fig. 1b).

### Delay of neurological reflexes maturation in HI rats

As it is shown in Fig. 1c, the right eye opening day was delayed in hypoxic-ischemic animals (*P* < 0.0001). In addition, several neurological reflexes, such as negative geotaxis, ear twitch reflex, auditory startle, hindlimb grasp, and gait reflex (*P* = 0.0042; *P* = 0.0025, *P* = 0.0032; *P* = 0.0127; *P* = 0.0008, respectively) appeared significantly later compared to normal pups. HI injury caused not only the delay in the appearance of some reflexes but animals performed certain tasks in significantly longer times (*data not shown*).

### Anxiolytic-like baseline behavior in males following HI injury

**Fig. 2:**
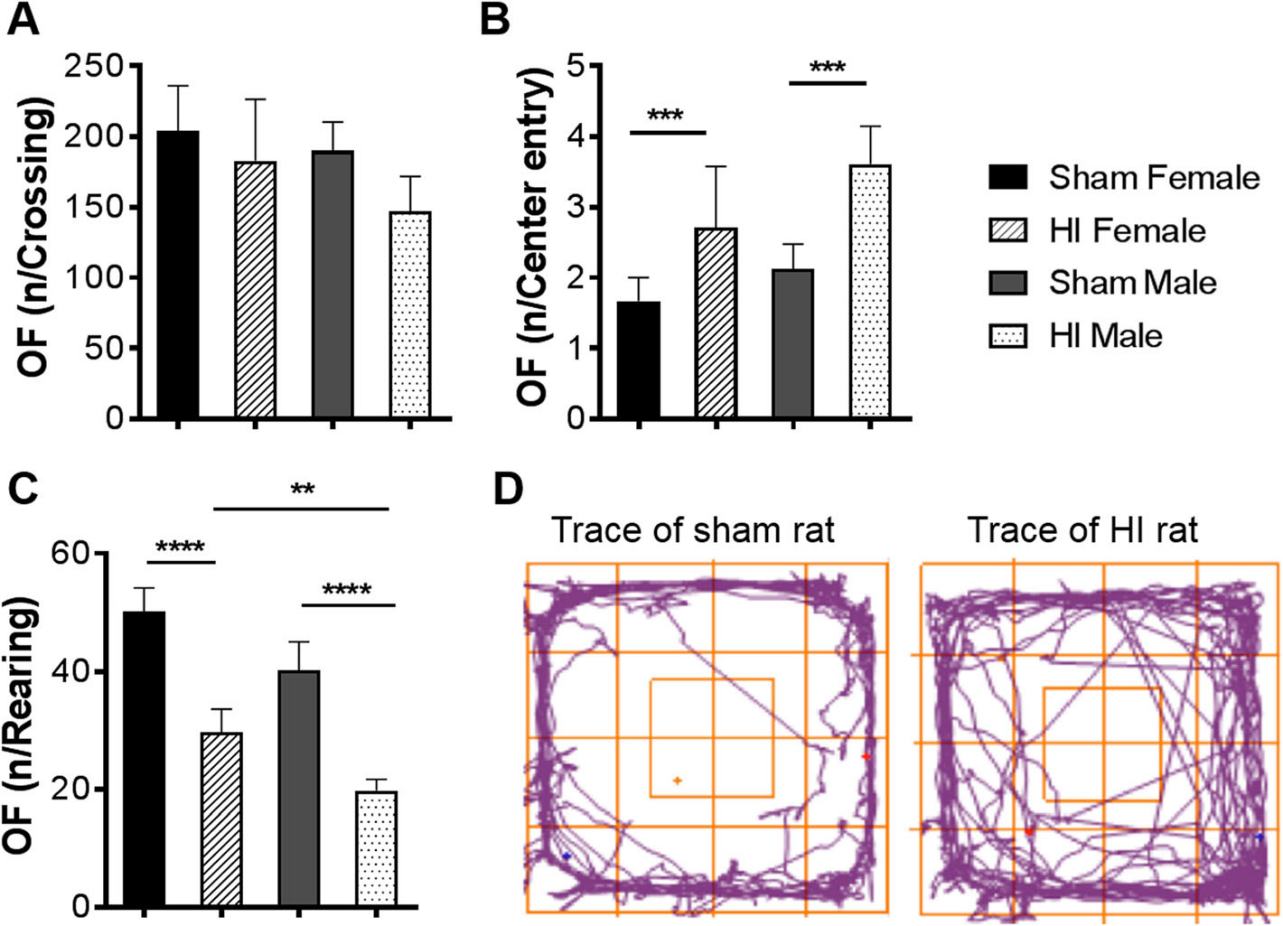
Effect of neonatal HI on open-field performance in sham and HI rats at P28*.* **a** Number of crossing. **b** Frequency of entry to the center. **c** Number of rearing. Graphs show average values ± S.E.M. **d** Representative traces of sham and HI rat movement during the open-field test. Statistical analysis: two-way ANOVA and multiple comparison test (**P < 0.01, ***P < 0.001, ****P < 0.0001). Test duration: 10 min. *N* = 13 male HI, 6 female HI, 8 male sham, 7 female sham. P, postnatal

The open-field test was performed in rats at P28 as a measure of anxiety response to novelty. There was no significant difference between HI and sham rats concerning the number of crossing, general activity, and movement pattern (Fig. 2a). HI males more than female rats spend significantly more time exploring the unprotected center area demonstrate anxiolytic-like baseline behavior (*P* = 0.0005; *P* = 0.0007, respectively) and less time in contact with the walls (thigmotaxis) comparing to sham rats (Fig. 2b, d). There was no significant difference in the time spent with grooming activity or in the number of fecal boluses at any time point between the different groups (*data not shown*) while a significant difference was observed in HI male and female rats in the number of rearing all along the test duration comparing to sham animals (*P* < 0.0001; *P* < 0.0001, respectively) as it is shown in Fig. 2c. Among HI rats, there was a significant difference between sexes (*P* = 0.0052).

### Cerebellar dysfunction following HI injury

**Fig. 3:**
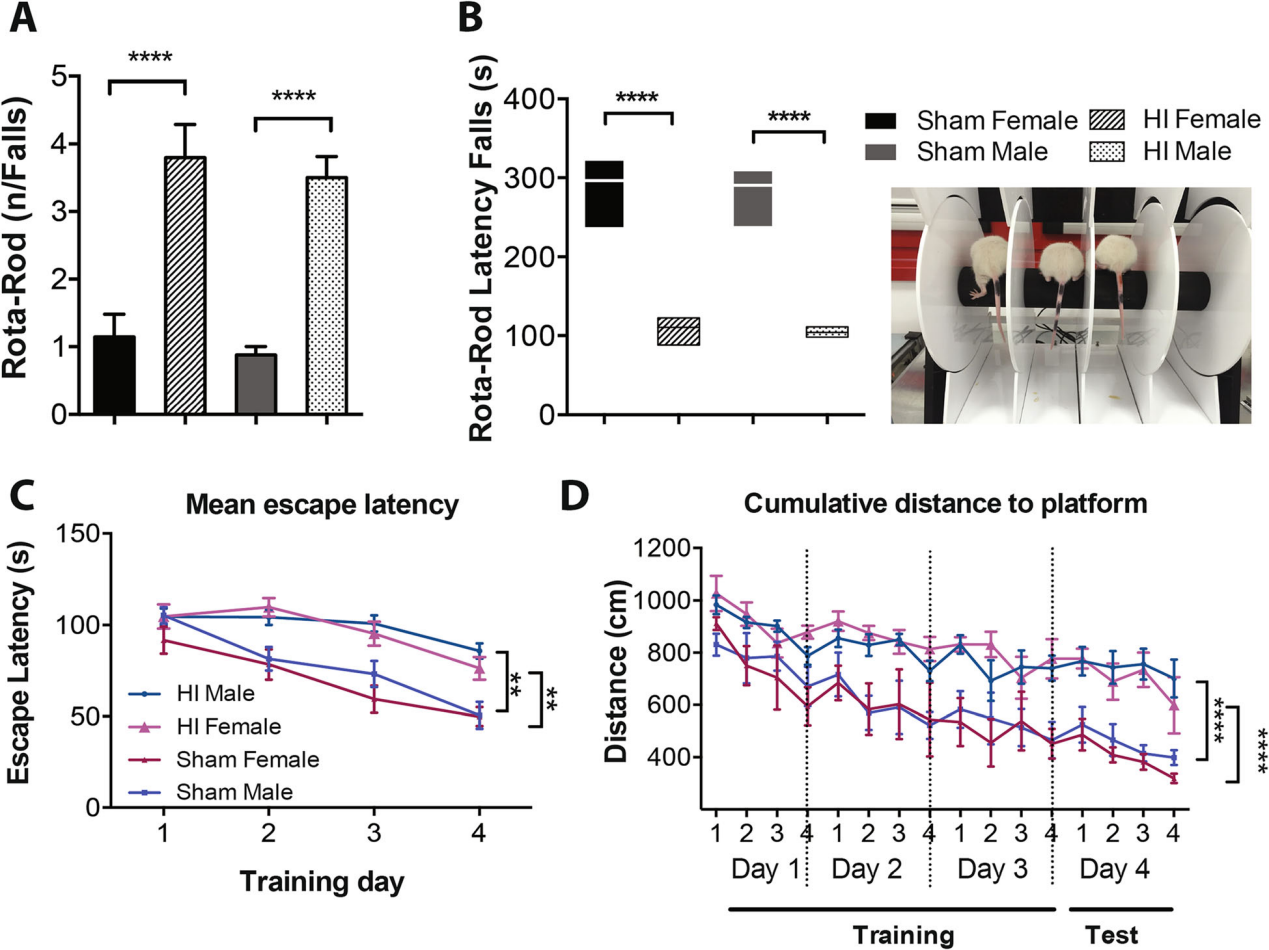
Effect of neonatal HI on rotarod and MWM performance. **a** Number of falls during the habituation session. **b** Latency of falls during the test session. Statistical analysis: two-way ANOVA and multiple comparison test. ****P < 0.0001. *N* = 13 male HI, 6 female HI, 8 male sham, 7 female sham. **c** Mean escape latency during the 4 days of training. **d** Cumulative distance to platform during the training and the test days. These tests were performed in rats at P28. Statistical analysis: two-way ANOVA and Tukey’s multiple comparison test. ****P < 0.0001. *N* = 13 male HI, 6 female HI, 8 male sham, 7 female sham

The rotarod test was performed in rats at P28 to evaluate cerebellum-dependent sensory-motor coordination. During the habituation session, HI (Fig. 3a) rats were not able to stay on the rod compared to sham when the rod was rotated at a steady rate of 20 rmp (*P* < 0.0001, *P* < 0.0001; HI female vs sham female and HI male vs sham male, respectively). During the test session (Fig. 3b), 24 h later, HI female and male rats were held on the rotarod for a significantly shorter time comparing to sham (*P* < 0.0001, *P* < 0.0001). Among HI rats, there were no significant differences between sexes.

### HI injury impairs spatial learning ability

In order to determine the effect of HI injury on cognitive capacities, 28-day-old rats were trained in the spatial version of the MWM. A two-way ANOVA test revealed significant differences in the escape latency of the second between the experimental groups (*P* < 0.0001, *P* < 0.0001; HI female vs sham female and HI male vs sham male, respectively) during the training period (Fig. 3c). As shown in Fig. 3d, the cumulative distance to the platform to 4 days and even on the test day was shorter in sham-operated rats when compared with the HI groups, indicating that HI impaired the memory performance in the injured animals. Among HI rats, no significant differences between females and males were detected.

### HI injury impairs locomotion coordination and gait

**Fig. 4:**
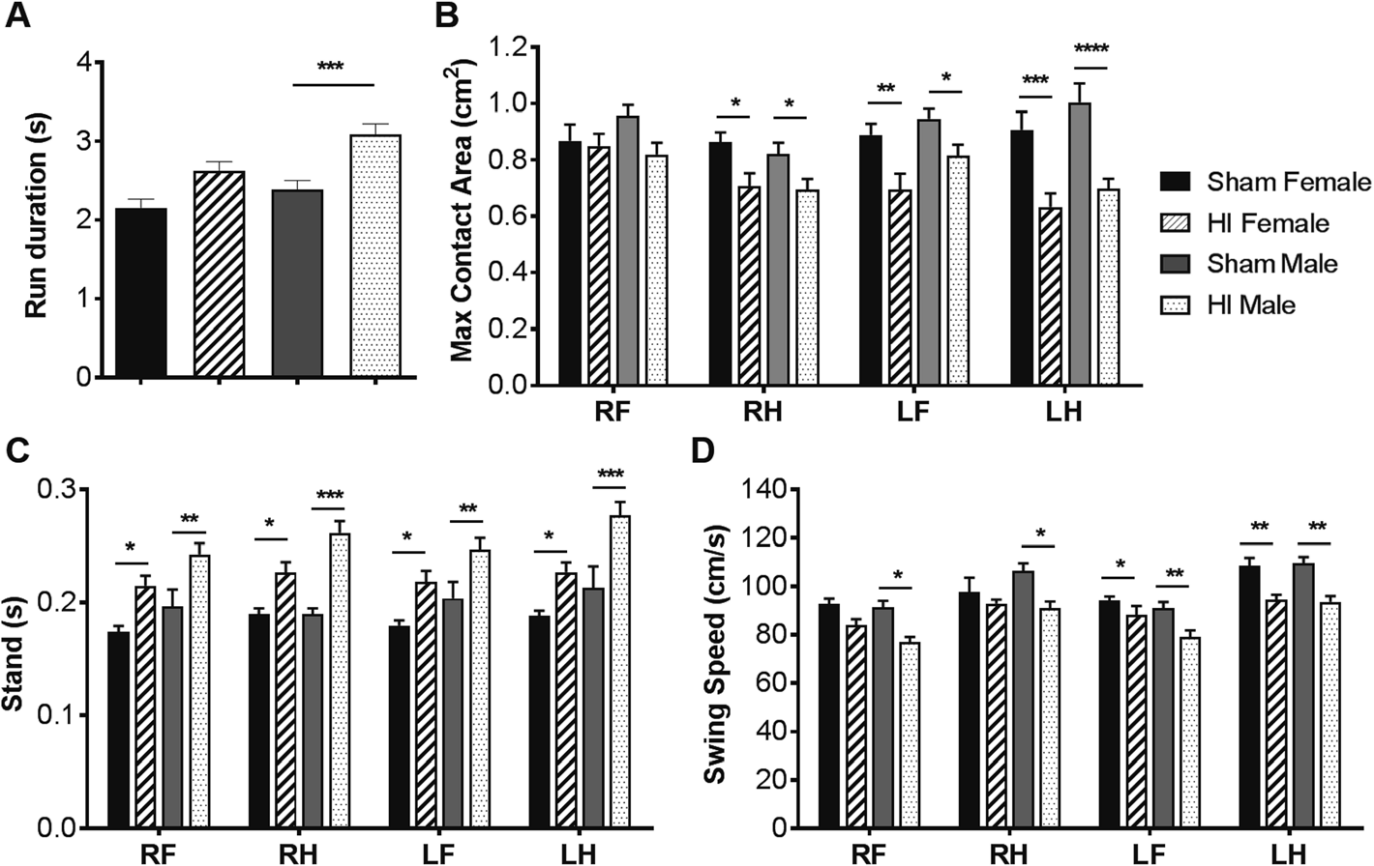
Motor function was assessed with a CatWalk gait analysis system at P28. **a** Run duration, **b** maximum contact area, and **c** stand and **d** swing speed. Statistical analysis: two-way ANOVA and Tukey’s multiple comparison test (*P < 0.05, **P < 0.01, ***P < 0.001, ****P < 0.0001). *N* = 13 male HI, 6 female HI, 8 male sham, 7 female sham. RF, right fore; RH, right hind; LF, left fore; LH, left hind limbs

The CatWalk assessment of motor function post HI in the rat demonstrates a long-term deficit in behavioral parameters related to the hind paw, in particular for the maximum contact area and stand and swing speed of the four limbs (Fig. 4). This test was performed in rats at P28. HI males have higher run duration than sham, (*P* < 0.001). Additional post Tukey analysis showed that HI animals have significant impairment in the maximum contact area of their right fore (RF), right hind (RH), left fore (LF), and especially the left hind (LH) paws comparing to sham rats (Fig. 4b) (*P* < 0.05, *P* < 0.01, *P* < 0.001, or *P* < 0.0001). Figure 4c indicates that the duration in seconds of contact of a paw with the glass plate of HI rats comparing with sham showed an increase in stand duration (*P* < 0.05, *P* < 0.01, or *P* < 0.001). In HI animals, the swing speeds (Fig. 4d) of their four limbs were all decreased compared to those of the rats in the sham group (*P* < 0.05 or *P* < 0.01). Overall, HI-induced sensorimotor function deficits in HI rats and HI males were worse than HI females.

### Alteration of the protein expression profile during the acute phase of HI

**Fig. 5:**
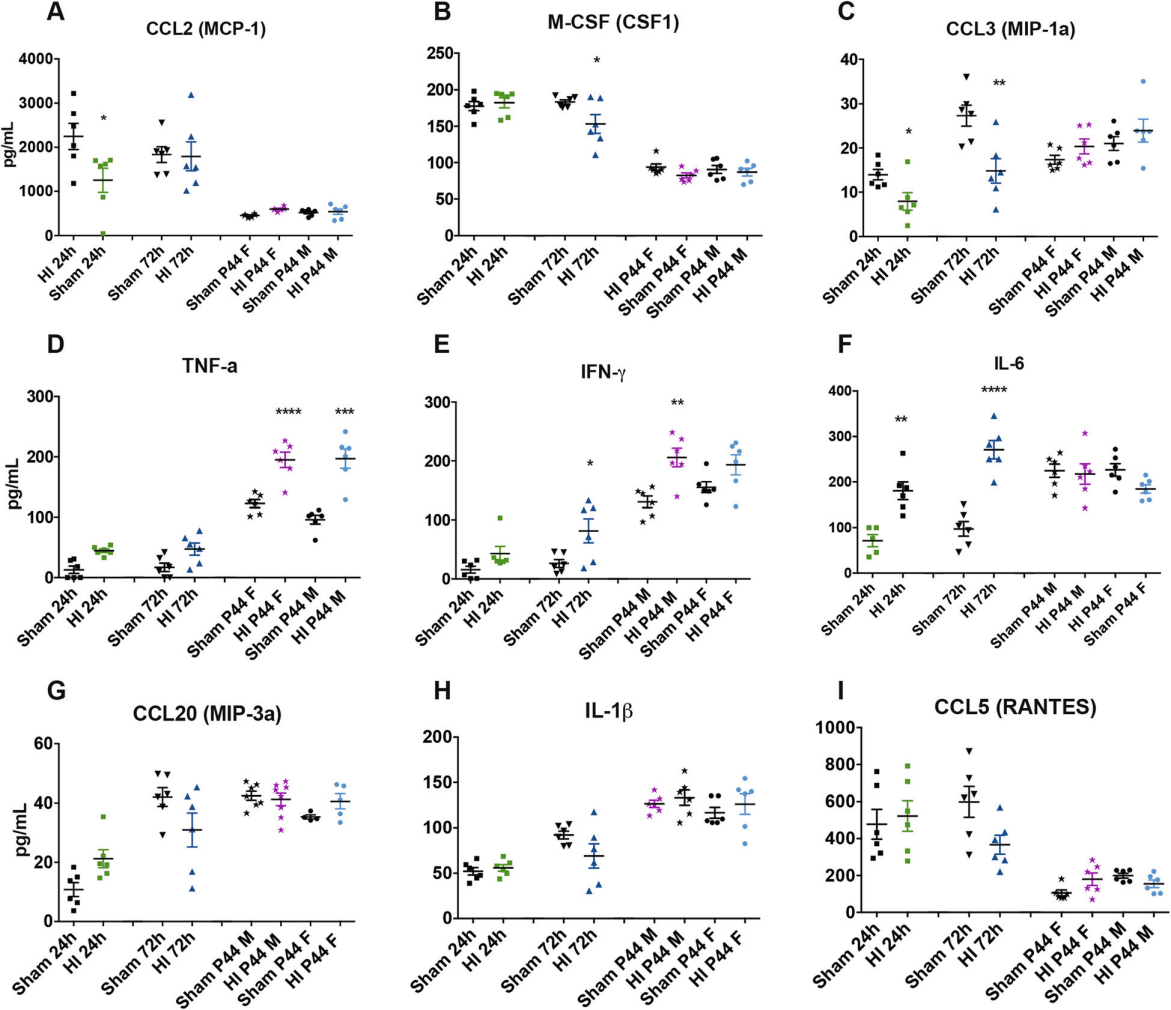
Alteration of the plasma protein expression after HI. The amount of **a** CCL2, **b** CSF-1, **c** CCL3, **d** TNF-α, **e** IFN-γ, **f** IL-6, **g** CCL20, **h** IL-1β, and **i** CCL5 in plasma in sham compared to HI groups after 24 h, 72 h, and 4 weeks in males and females is reported. Results are presented as individual values (picograms/milliliter) and the mean ± SEM is also shown. Statistical analysis: 24 h HI (*n* = 6), 24 h sham (*n* = 6), 72 h HI (*n* = 6), 72 h sham (*n* = 6), P44 HI male (*n* = 6), P44 sham male (*n* = 6), P44 HI female (*n* = 6), P44 sham female (*n* = 6), one-way ANOVA, and Tukey’s multiple comparison test (*P < 0.05, **P < 0.01, ***P < 0.001, ****P < 0.0001)

**Fig. 6:**
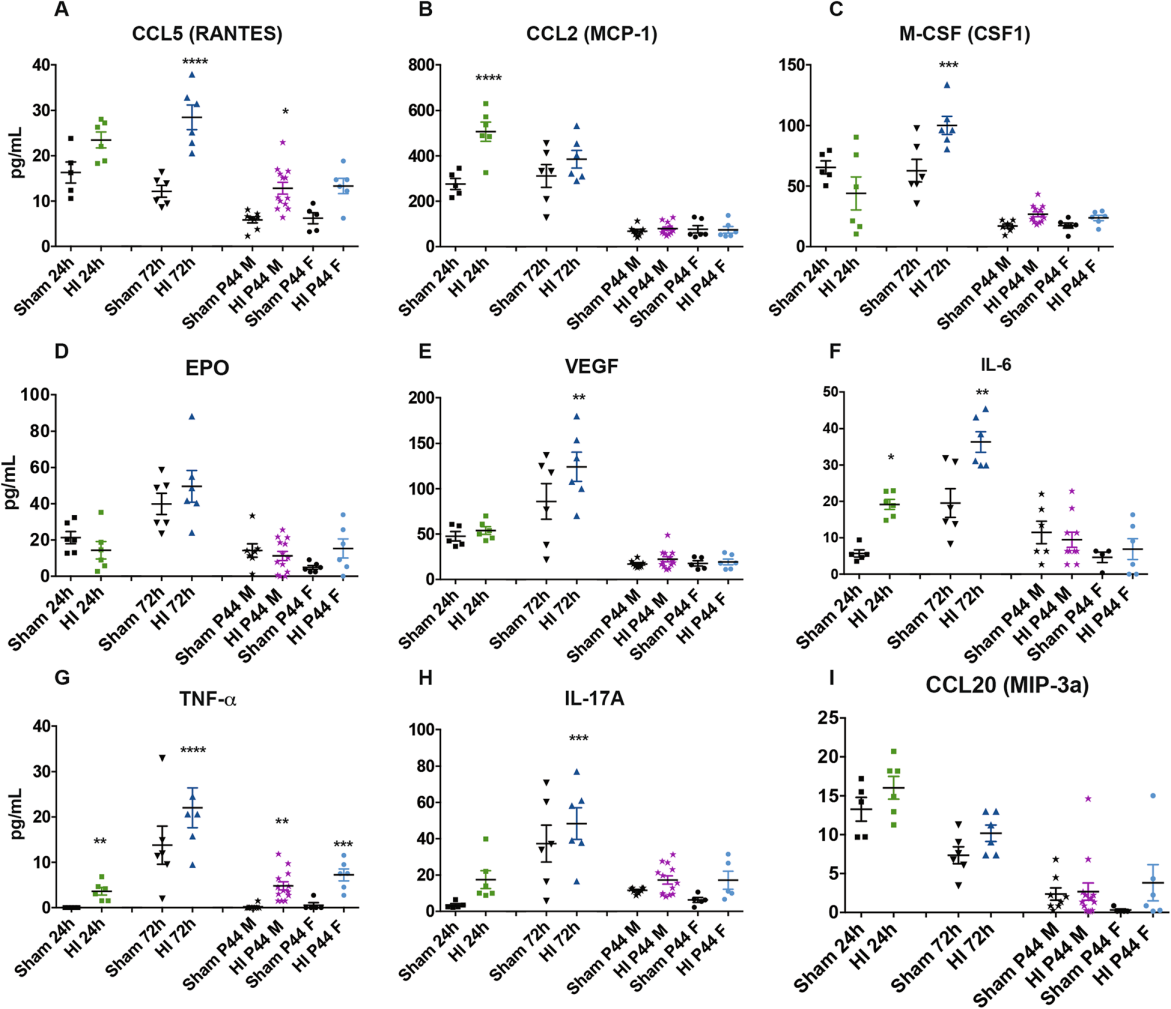
Alteration of the CSF protein expression after HI. The amount of **a** CCL5, **b** CCL2, **c** CSF1, **d** EPO, **e** VEGF, **f** IL-6, **g** TNF-α, **h** IL17-A, and **i** CCL20 in CSF in sham compared to HI groups after 24 h, 72 h, and 4 weeks in males and females is reported. Results are presented as individual values (picograms/milliliters) and the mean ± SEM is also shown. Statistical analysis: 24 h HI (*n* = 6), 24 h sham (*n* = 6), 72 h HI (*n* = 6), 72 h sham (*n* = 6), P44 HI male (*n* = 6), P44 sham male (*n* = 6), P44 HI female (*n* = 6), P44 sham female (*n* = 6), one-way ANOVA, and Tukey’s multiple comparison test (*P < 0.05, **P < 0.01, ***P < 0.001, ****P < 0.0001)

Twenty-four cytokines and chemokines were simultaneously quantified in plasma and CSF samples at the different investigated time points, in the acute phase, e.g., 24 and 72 h and in the chronic phase, e.g., 4 weeks. Several proinflammatory biomarkers such as CCL2, CCL3, and IL-6 (*P* = 0.0184, *P* = 0. 0337, and *P* = 0.0020, respectively) in plasma (Fig. 5a, c, and f and Additional file 3: Table S1) and TNF-α and CCL2 (*P* = 0.0412; *P* < 0.0001, respectively) in CSF (Fig. 5g, b and Additional file 4: Table S2) were significantly regulated as soon as 24 h after HI while the major part of the biomarkers investigated was regulated 72 h after HI such as CSF1, IFN- γ, and CCL5 (*P* = 0.0483; *P* = 0.0193; ns, respectively) in plasma (Fig. 5b, e, and i and Additional file 3: Table S1) and CCL5, CSF1, VEGF, and IL-17A (*P* < 0.0001; *P* = 0.0003; *P* = 0.0024, *P* < 0.0001, respectively) in CSF (Fig. 6a, c, e, and h and Additional file 4: Table S2).

During the chronic phase, 4 weeks after the injury, in plasma, only TNF-α and IFN-γ (*P* < 0.0001; *P* = 0.0047, respectively) were significantly upregulated at this time point in male HI compared to sham male (Fig. 5d, e and Additional file 3: Table S1) and only TNF-α (*P* = 0.0009) was significantly increased on female HI compared to the female sham group (Fig. 5d and Additional file 3: Table S1). In CSF, only CCL5 and TNF-α (*P* = 0.0164; *P* = 0.0012, respectively) were significantly upregulated at this time point in male HI compared to sham male (Fig. 6a, g and Additional file 4: Table S2) and only TNF-α (*P* = 0.0164) was significantly increased on female HI compared to the female sham group (Fig. 6g and Additional file 4: Table S2). The rest of the measurable biomarkers of the panel, at the chronic phase in plasma and CSF HI rats, showed no significant change neither between HI and sham males nor between HI and sham females. Among HI rats, there were no significant differences between sexes (Figs. 5 and 6 and Additional file 3: Table S1 and Additional file 4: Table S2).

No significant changes were observed for the rest of the panel compared to the sham group. The biomarkers G-CSF (CSF3), GM-CSF (CSF2), IL-1α, IL-7, IL-10, and IL-12p70 were not detected at any of the time points analyzed in the CSF in our experimental conditions.

### Altered gene expression profile induced by HI

**Fig. 7:**
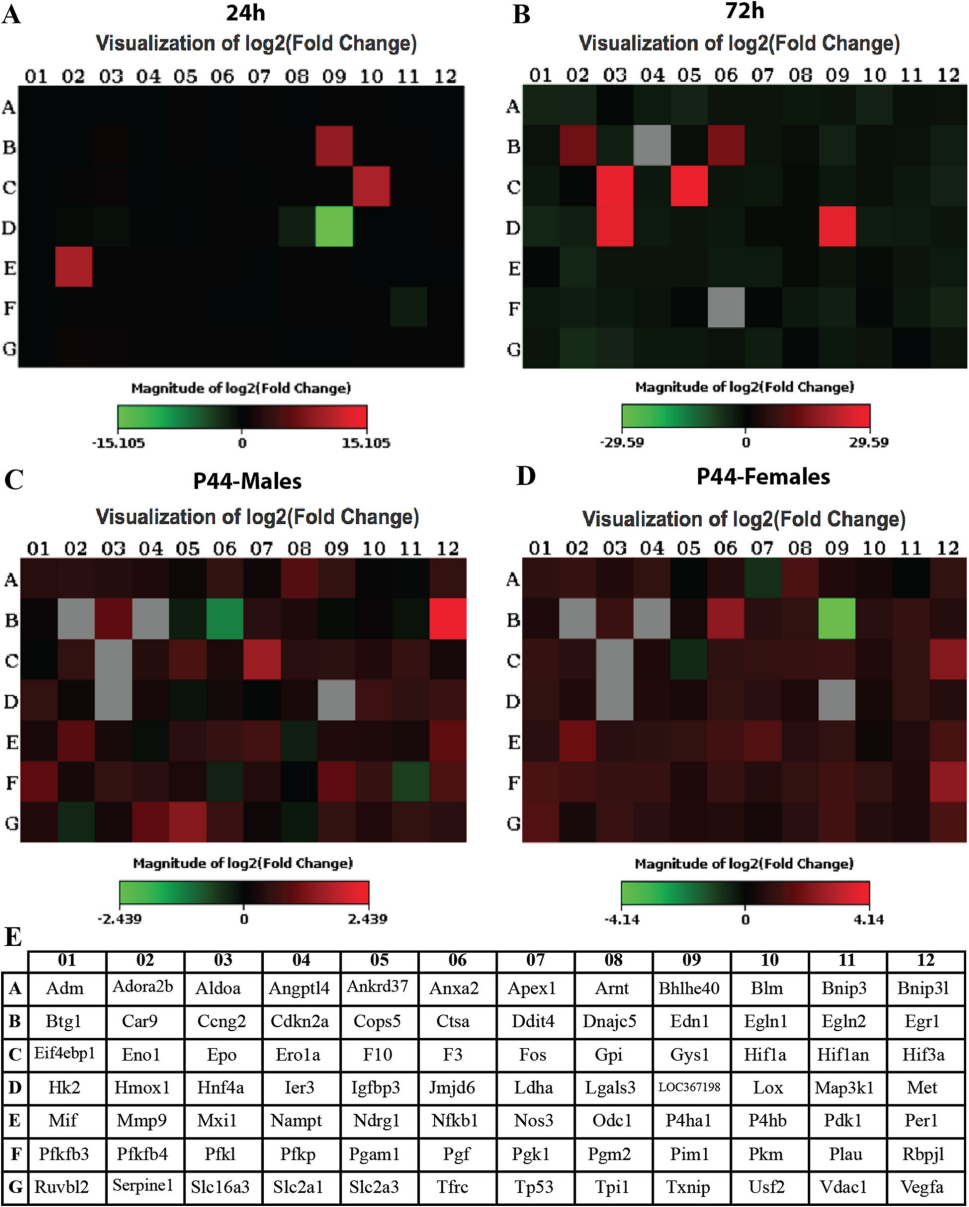
Alteration of the gene expression profile after HI*.* Gene expression profile in the ipsilateral vs contralateral hemisphere of rats induced by hypoxia-ischemia after 24 h (**a**), 72 h (**b**), and 4 weeks in males (**c**) and females (**d**). Results are represented as log2-fold change in a heat map. Red color denotes upregulated genes and green color downregulated genes; black represents unchanged gene expression and the gray color slightly upregulated gene. Statistical analysis was performed using Student’s *t* test of the replicate 2^−∆Ct^ values for each gene in the control group and treatment group. **e** Complete list of investigated genes by RT^2^ Profiler™ PCR Array

The mRNA expression of genes directly involved in the response to hypoxia and oxidative stress, apoptosis, signal transduction, and protein metabolism, as well as genes involved in cell growth and metabolism, extracellular matrix, and adhesion molecules, was studied in the ipsilateral vs the contralateral hemisphere of both HI and sham animals by real-time PCR. The complete list of investigated genes is presented in Fig. 7e. At 24 h post-injury, Edn1, Hif1-α, and Mmp9 were highly upregulated in the ipsilateral vs the contralateral hemisphere of hypoxic-ischemic rats (447.65 log2-fold, 169.20 log2-fold change, 103.67 log2-fold change, respectively). LOC367198 was downregulated (− 30.38 log2-fold change) (Fig. 7a, e and Additional file 2: Figure S2A, E). At 72 h post-injury, Car9, Epo, Hnf4a, F10, and Cdkn2a were highly upregulated in the ipsilateral vs the contralateral hemisphere of hypoxic-ischemic rats (106.97 log2-fold change, 182.27 log2-fold change, 110.43 log2-fold change, 309.23 log2-fold change, and 32.64 log2-fold change, respectively) (Fig. 7b, e and Additional file 2: Figure S2B, E). Most of the genes were upregulated in the ipsilateral vs the contralateral hemisphere of HI rats (both sexes) during the chronic phase (4 weeks after the injury), in particular, Egr-1. Ctsa was downregulated (− 2.35 log2-fold change) for HI male rats (Fig. 7c, e and Additional file 2: Figure S2C, E) and Edn1 (− 17.63 log2-fold change) for HI female rats (Fig. 7d, e and Additional file 2: Figure S2D, E).

## Discussion

The main goal of the present study was to investigate potential plasma and CSF biomarkers of brain damage after acute and chronic HI, and also according to the neurological maturation and development of complex behaviors. Our results point out the potential critical role of inflammatory biomarkers to enable early identification of infants at risk of long-term injury but also for monitoring treatment efficacy that can produce significant and lifelong improvements on the quality of life for neonates.

HI was induced at P7 in both female and male Wistar rats. We have shown that, in both female and male rats, HI leads to tissue damage and atrophy of the hypoxic hemisphere that is accompanied by short-term, retarded neurobehavioral development (delayed appearance and worse performance of some neurological reflexes), as well as long-lasting deficits in complex behaviors (retarded development of motor coordination and impaired learning in spatial tasks).

We first characterized the neurological and behavioral maturation of HI compared to sham-operated rats. Although it was previously reported that in this HI model, pups can suffer of diminished weight gain during the development and thus needing artificial feeding [43, 44], in our HI rat model, we did not observe any significant difference in the somatic development. This is probably because we pre-established the study design stringent inclusion/exclusion criteria related to the bodyweight of the pups, and this could have improved the health status, survival, and experimental variability. Our results show that HI animals perform worse in negative geotaxis, ear twitch, auditory startle, hindlimb grasp, and gait test as measured by the reflex times, independent of sex. This is in line with previous studies showing that HI in rodent models affects the short-term outcome of righting, geotaxis reflexes as measured 1 and 24 h after the insult [30, 43, 45]. We extended our observations to 4 weeks post-injury and found that some neurological reflexes are impaired also in the chronic phase.

In order to assess the long-term neurofunctional consequences following neonatal HI, several complex behaviors have been examined in 6-week-old animals, including sensorimotor integration in accelerating rotarod, spontaneous locomotion in open field, gait in CatWalk apparatus, and spatial learning in Morris water maze.

Concerning the locomotor behavior in an open field, both hyper- and hypoactivity have been described following HI insults. In accordance with other reports [34, 45, 46], we did not see any significant differences concerning the number of crossing, general activity, and movement patterns in the time spent with grooming activity or in the number of fecal boluses at any time point between the different groups. However, a significant decrement was observed in HI rats (both males and females) in the number of rearing in comparison to sham animals. In adult rats (15 weeks old), hypoactivity has been described [47]. We observed also that both HI female and male rats spent more time in the center and less time at walls and in corners than sham rats, which is exactly the opposite of the natural reflex of the pups, who quickly find the wall where they feel safer.

In order to determine the effect of HI insult on cognitive capacities, particularly in the spatial learning and memory, the MWM test was used. The following parameters were considered: the escape latency to reach the platform; a revealing parameter of working memory, which is tested during the training phase; and the reference memory components recorded on the probe phase (fifth day) of the task, including the cumulative distance to platform. Our data clearly indicates that both learning and memory performance are affected in the injured animals. Our findings are in agreement with results from De Paula et al. and Goren et al., showing that the MWM test was sensitive to brain damage in neonatal HI rats [48, 49].

The CatWalk assessment of gait demonstrates the long-term deficit in several parameters related to the hind paws of HI compared to sham-operated rats. In particular, HI animals showed a significant impairment in the maximum contact area of their four limbs, especially the left hind paws, in comparing to sham rats. In addition, the duration of contact of a paw with the glass plate showed an increase in stand duration in HI rats compared with sham. In addition, the swing speed of all four limbs was decreased in HI rats. Overall, HI males performed worse than HI females, a finding in line with the notion that important cerebral palsy and related developmental disorders are more common in males than in females [50]. Data from neonatal rodents subjected to hypoxia-ischemia also demonstrate the involvement of different death pathways between males and females. For instance, the knockout of the gene for poly (ADP-ribose) polymerase (PARP-1), a major step in the cascade of injury, protected male but not female mouse pups from hypoxic-ischemic injury [51].

Interestingly, HI insult does induce sensorimotor functional deficits in some parameters while in others, we observe a slight recovery and no significant difference between HI and sham group. This could be explained by the extraordinary plasticity of the neonatal brain, allowing compensatory mechanisms such as the shift of motor coordination control from a damaged brain area to an unharmed one [34, 49, 52].

We then investigated several potential biomarkers of brain damage in plasma and CSF, considering that this model includes systemic hypoxia and as such, much of the increases in cytokines in the blood could well be coming from systemic organ damage [2, 53, 54]. Several studies have shown that neonatal HI triggers widespread inflammatory reactions in the brain including the activation of the innate immune system [55, 56]. We focused on related cytokines and chemokines, using a Luminex-based multiparametric assay. Our results demonstrate that an early regulation of most inflammatory biomarkers was observed as soon as 24 and 72 h, after HI, while no significant change neither between HI and sham males nor between HI and sham females was observed at the chronic phase, in both plasma and CSF. Some proinflammatory biomarkers such as CCL3, CCL2, and IL-6 in plasma and CSF regulated as soon as 24 h after HI while most of the biomarkers measured, such as CSF1, IFN- γ, CCL5, were regulated 72 h after HI. Notably, it has been shown that in term neonates, the CSF levels of IL-6 after perinatal asphyxia correlates to the severity of early neonatal HI and to late neurological outcome [15, 56–60]. In our model, we do observe an upregulation of IL-6 at 24 and 72 h in plasma and CSF. Both CCL2 and CCL3 are necessary for recruiting monocyte to the injury site, where they play an important role in CNS plasticity and repair [61, 62]. It was recently described that CCL2 and its receptor CCR2 regulate macrophage trafficking by induction of leukocyte adhesion to the microvascular endothelium after brain injury [63]. In our model, 24 h after HI, we observed a decrease in the plasma level of CCL2 and CCL3 with concomitant upregulation in CSF. This may reflect an early rapid response of the immature brain to the insult aimed at recruiting cells to the injury site. Schilling et al. have demonstrated that CCL2-CCR2 axis differentially regulates hematogenous cell recruitment and sequential inhibition of selective CCL2-dependent pathways by CCR2 blockade may be an effective treatment to ameliorate tissue damage [63].

We also show that TNF-α was upregulated in CSF as soon as 24 h after HI till the end of the experiment while in plasma, it was upregulated only during the chronic phase. It has been demonstrated previously that TNF-α is mainly secreted by activated microglia and can activate death receptors, such as TRAIL, on neurons and oligodendroglia [64]. In addition, TNF-α can potentiate glutamate-mediated cytotoxicity by two mechanisms: indirectly, by inhibiting glutamate transport on astrocytes, and directly, by rapidly triggering the surface expression of Ca (+ 2) permeable AMPA receptors and NMDA receptors, while decreasing inhibitory GABAA receptors on neurons [65]. Thus, TNF-α may contribute to the tissue damage and loss that was observed at sacrifice. Moreover, TNF-α plays a critical role in HI by inducing neutrophil infiltration, increasing the permeability of endotheliocyte and activating matrix metalloproteinases, which damage the blood-brain barrier (BBB) leading to swelling and degeneration of neurons and glial cells [23, 66]. Kaur et al. have demonstrated that the unmyelinated axons showed an upregulated expression of TNF-R1 coupled with the disruption of myelin basic protein immunopositive processes of oligodendrocytes in the periventricular white matter of HI neonatal rats suggesting that an overproduction of TNF-α may damage axons and delay their myelination [22, 67]. It has been shown that an increased level of TNF-α and INF-γ in the blood plays a role in depression- and anxiety-like behaviors [68, 69] which may explain the significant results that we observed in the open-field task with HI rats comparing to sham.

We also observed an increase in CSF1 (macrophage colony-stimulating factor) in HI rats. In particular, CSF1 was significantly increased in the acute phase after HI insult, at 72 h, in plasma and in CSF. We have previously suggested in EAE (experimental allergic encephalomyelitis), a rat model for multiple sclerosis, that the overexpression of CSF1 (macrophage colony-stimulating factor) in CSF aggravate the inflammatory process by propagating the proinflammatory signals to the nearby resting microglia and astrocytes through an increased production of proinflamamtory cytokines [70]. This possibility was also investigated on the HI rodent model and recent results suggest that amoeboid microglial cells derived from CSF1 promotes astrocytes to generate proinflammatory cytokines, which may be involved in axonal damage following HI insult [22, 67, 71–73]. According to our results, it could thus be speculated that CSF1 signaling plays an important role in the early phase of HI, by trigging microglial activation, subsequent induction of neuroinflammation, and axonal damage, and this could lead to short-term as well as long-lasting behavioral deficits after the injury. Therefore, following HI, a synchronized inflammatory response in the brain arises that makes a significant contribution to HI-induced neuronal death.

Notably, inflammatory biomarkers show a gender-related difference at early and late stages in both plasma and CSF, as already described for TNF-α [74], possibly reflecting the well-known gender-dependent immune and inflammatory responses described in several human inflammatory diseases [75] and in preclinical settings [76–78]. Overall, our data indicated the plasma biomarkers and not just CSF, as soon as fully characterized, and might as well monitor ongoing brain pathology.

We finally explored mRNA expression level in the cerebral cortex for several (*N* = 80) genes involved in tissue response to hypoxia. In particular, we found that Edn1, Hif1-α, and Mmp9 were strongly upregulated in the ipsilateral lesion—compared to the contralateral hemisphere in HI rats at 24 h post-injury. It is known that endothelin-1 (ET-1) is a peptide hormone, encoded by Edn1 gene, with potent vasoconstrictor properties and is commonly involved in ischemia/hypoxia-associated microvascular endothelium [38, 79, 80]. Indeed, ET-1 peptide and mRNA levels are upregulated upon HI insult in mice [81]. Tsang et al. also found that ET-1 mRNA levels in the astrocyte-like cells and vascular endothelial cells are dynamically regulated by ischemia and may participate in perinatal ischemia-related neural damage [82].

ET-1 as a drug efficacy biomarker is well illustrated by the pre-clinical studies supporting the use of vasopressin in neonatal revitalization. This treatment has a neuroprotective potential as several studies demonstrated that vasopressin activates hippocampal interneurons, silencing synchronous neuronal activity [83]. This could reduce neuronal energy demand, which might have a neuroprotection outcome. Therefore, the detection of ET-1 as an inflammatory biomarker not only for early diagnoses but also for monitoring treatment efficacy and as prognostic for recovery and long-term disabilities could have beneficial outcomes.

Edn1 is regulated by Hif1-α [84, 85] and the lack of this anti-survival factor in Hif1-α deficient mice might protect from early acute neuronal cell death and neurological impairment in the very acute phase after ischemic stroke [86]. It is also known that Hif-1α upregulation mediates BBB damage in acute cerebral ischemia [87–89]. The matrix metallopeptidase 9 (MMP9), when activated upon both cerebral ischemia and inflammation, can degrade collagen type IV in the endothelial basement membrane, leading to disruption of the BBB and allowing entry of peripheral immune cells into the brain [21, 79, 90–94]. Taking together, our results suggest that the response to HI injury occurs as soon as 24 h post-injury with an increase in Edn1, Hif1-α, and Mmp9, which may affect the cerebral blood fluid flow, the BBB properties, and the infiltration of the peripheral immune cells into the brain. At 72 h post-HI injury, we found that Car9, Epo, Hnf4a, F10, and Cdkn2a mRNA levels were highly upregulated in the ipsilateral vs the contralateral hemisphere, suggesting the rapid follow-up of the nervous system of the injury occurred at 24 h and the attempt to recover. Those genes are involved in the clotting and hematopoietic processes, such as factor X (F10) gene that code for coagulation factors [95, 96] and Epo gene that has been known to stimulate the erythropoiesis in response to cellular hypoxia [97–99]. Interestingly, EPO is known to promote neurorestoration upon HI insults, and thus, the delayed EPO upregulation observed in this study could reflect a recovery attempt of the brain. EPO is an endogenous protein, synthesized in the fetal liver that has an impact on multiple critical pathways and influences the body’s immune response [100]. EPO and EPO receptor are upregulated following HI injury and EPO has an anti-oxidant [101] as well as an anti-inflammatory [102] effect. It reduced apoptotic and excitotoxic cell injury [103–105]. Clinical trials evaluating EPO in infants with HI have shown to be promising; since hypothermia has become a standard of care therapy for HI, larger trials are currently ongoing evaluating EPO as a complement to cooling therapy results [106, 107].

During the chronic phase, i.e., 4 weeks after the HI injury, most of the genes were still upregulated in the ipsilateral vs contralateral hemisphere in both male and female rats. These findings might suggest that despite the attempt of recovery in the acute phase, as discussed earlier, the consequences of the early activation of the inflammatory cascade lead to extensive brain hemisphere atrophy which explains long-lasting behavioral deficits. Comparing to females, male HI rats have increased the mRNA level of Erg-1 (nerve growth factor-induced protein A). It has been shown that chemokine, adhesion receptor, procoagulant, and permeability-related genes are coordinately upregulated by rapid ischemia-mediated activation of Egr-1 [108, 109]. This might correlate with the impaired long-term memory observed in our model at the chronic phase. Indeed, Erg-1 has a critical role in the context of neuropsychiatric disorders due to its involvement in critical processes underlying neuronal activity, from neurotransmission and synaptic plasticity, to higher-order processes such as learning and memory, response to emotional stress and reward [110]. Jones et al. also showed that Egr-1 knockout mice have impaired spatial long-term memory while the short-term spatial memory is intact, suggesting a critical role for EGR1 in memory consolidation [111].

Ctsa (cathapsin A) mRNA level was downregulated in HI male at the chronic phase which might correlate with the decline in cognitive functions as well as in learning and memory observed in HI males comparing the females. A recent study showed that *CathA*^*S*190*A*^ mice (mutant mouse with catalytically inactive cathapsin A enzyme) have learning impairments as well as long-term and spatial memory deficits compared with wild-type littermates, suggesting that Ctsa plays a significant role in learning and in memory consolidation [112].

While either sex of rats has been used for modeling hypoxia seizures [113], male rats are preferred in most studies to avoid potential bias due to sex differences [114]. Female rats show different developmental GABA profile during the critical period [115] and therefore respond differently to hypoxia-induced neonatal seizures. In our model, HI leads to an equivalent level of primary brain injury in males and in females during the acute phase (24 and 72 h after injury), no differences in the gene and protein expression. However, we observed a significant difference between male and female HI rats in the open field and the CatWalk tasks were males performed worse than females. Mirza et al. also previously reported this, where they showed that HI female mice have less behavioral deficits compared to males at the chronic stage of HI [116]. It is believed that HI is increasingly recognized as a sexually dimorphic disease where male infants suffer more long-term cognitive deficits compared with females with comparable brain damage [74, 116, 117]. In a more recent study done by Barkhuizen et al., where the long-term efficacy of multipotent adult progenitor cells was investigated, they found a persistent cognitive rescue in the female rats compared to the males. The gender differences they observed in the therapeutic responses are indicative of the clinical scenario where male infants have more mortality and morbidity after encephalopathy than females [118].

## Conclusion

In conclusion, in our model of HI brain injury in juvenile rats, we have shown an early activation of the inflammatory cascade leading to increased production of several proinflammatory cytokines and chemokines such as TNF-α and IL-6. These early responses could contribute to a cascade of events causing delayed cellular death and the subsequent tissue damage occurring in the hemisphere subjected to HI. This cascade of events, in turn, leads to the short-term as well as long-lasting neurobehavioral deficits, such as retarded development of motor coordination, observed in this study. Since cytokine, such as TNF-α, production precedes and can contribute to brain damage, their significance as early biomarkers is relevant. A full characterization of the early neuroinflammatory responses could be of diagnostic and prognostic value and also for initiating and monitoring current and future therapeutic intervention aimed to neuroprotection.

## Supplementary information

**Additional file 1: Figure S1**. *Timeline of the experimental procedure and behavioral tests performed after HI.* HI was induced at P7 in both female and male Wistar pup rats (12-14 g weight). Two animal groups were sacrificed 24 and 72 h after injury. A third group was subject to behavioral tests: from P8 to P21 neurological reflex tests were performed. The same group was subject to the different long-term behavioral test (open field, Rota-rod, catwalk, and MWM) with one or 2 days off between the tests. At the end of the final test (MWM) rats were sacrificed for transcriptomic and proteomic analysis.

**Additional file 2: Figure S2.** Log2 fold change of gene expression in the ipsilateral vs contralateral hemisphere of rats induced by Hypoxia ischemia after 24 h (A) 72 h (B) 4 weeks in males (C) and females (D).

**Additional file 3: Table S1.** Raw data of plasma level of different cytokines, chemokines and growth factors in sham and HI rats at different time points. Results are presented as mean ± SEM (pg/mL), with the *P* value (one-way ANOVA and Tukey’s multiple comparison test).

**Additional file 4: Table S2.** Raw data of CSF level of different cytokines, chemokines and growth factors in sham and HI rats at different time points. Results are presented as mean ± SEM (pg/mL), with the *P* value (one-way ANOVA and Tukey’s multiple comparison test).

## Data Availability

The datasets used and analyzed during the current study are included within the article and its additional files. All material used in this manuscript will be made available to researchers subject to confidentiality.

## Abbreviations

BBB: Blood-brain barrier
CCA: Common carotid artery
CNS: Central nervous system
CSF: Cerebral spinal fluid
CT: Computed tomography
EEG: Electroencephalogram
HI: Hypoxia-ischemia
MRI: Magnetic resonance imaging
MWM: Morris water maze
