# Supplementary figures and images for: Potential biomarkers for neuroinflammation and neurodegeneration at short and long term after neonatal hypoxic-ischemic insult in rat
Source: J Neuroinflammation. 2019 Oct 28;16:194. doi: 10.1186/s12974-019-1595-0 (PMC6819609; doi:10.1186/s12974-019-1595-0)

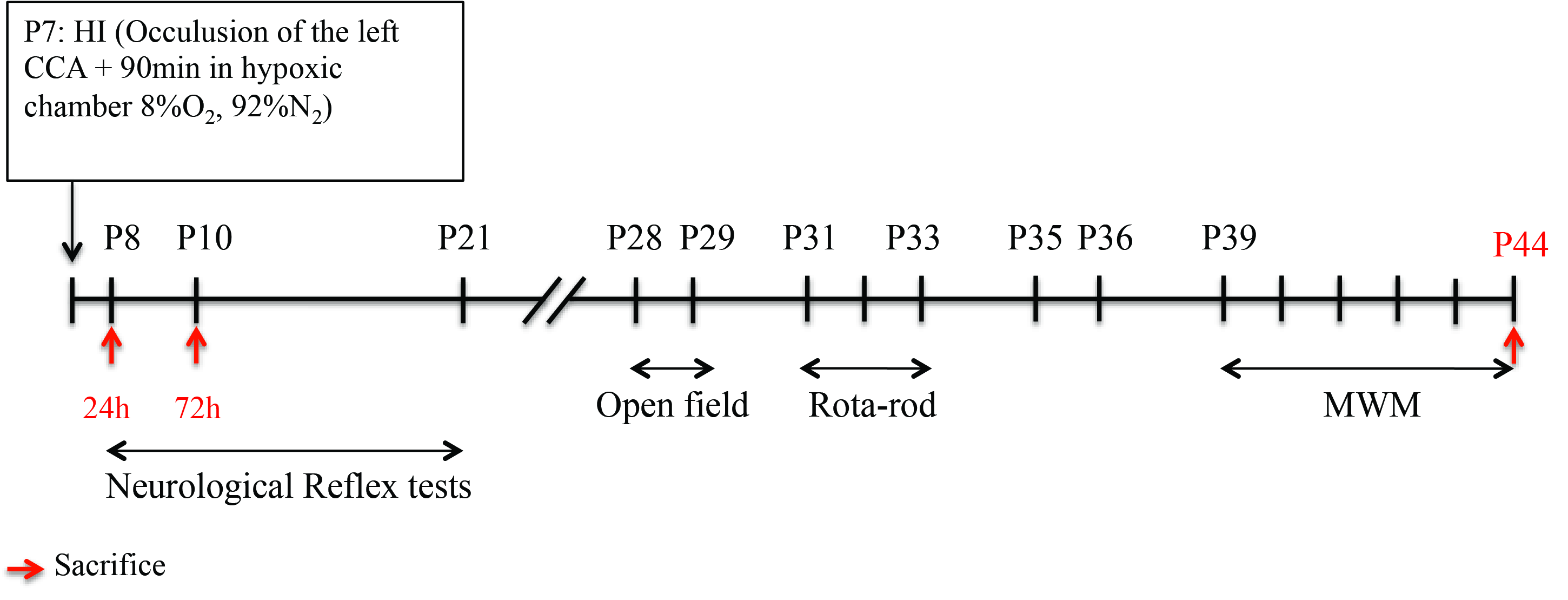

Supplement: Supplementary file 1 — Additional file 1: Figure S1. Timeline of the experimental procedure and behavioral tests performed after HI. HI was induced at P7 in both female and male Wistar pup rats (12-14 g weight). Two animal groups were sacrificed 24 and 72 h after injury. A third group was subject to behavioral tests: from P8 to P21 neurological reflex tests were performed. The same group was subject to the different long-term behavioral test (open field, Rota-rod, catwalk, and MWM) with one or 2 days off between the tests. At the end of the final test (MWM) rats were sacrificed for transcriptomic and proteomic analysis. [file 12974_2019_1595_MOESM1_ESM.tif]

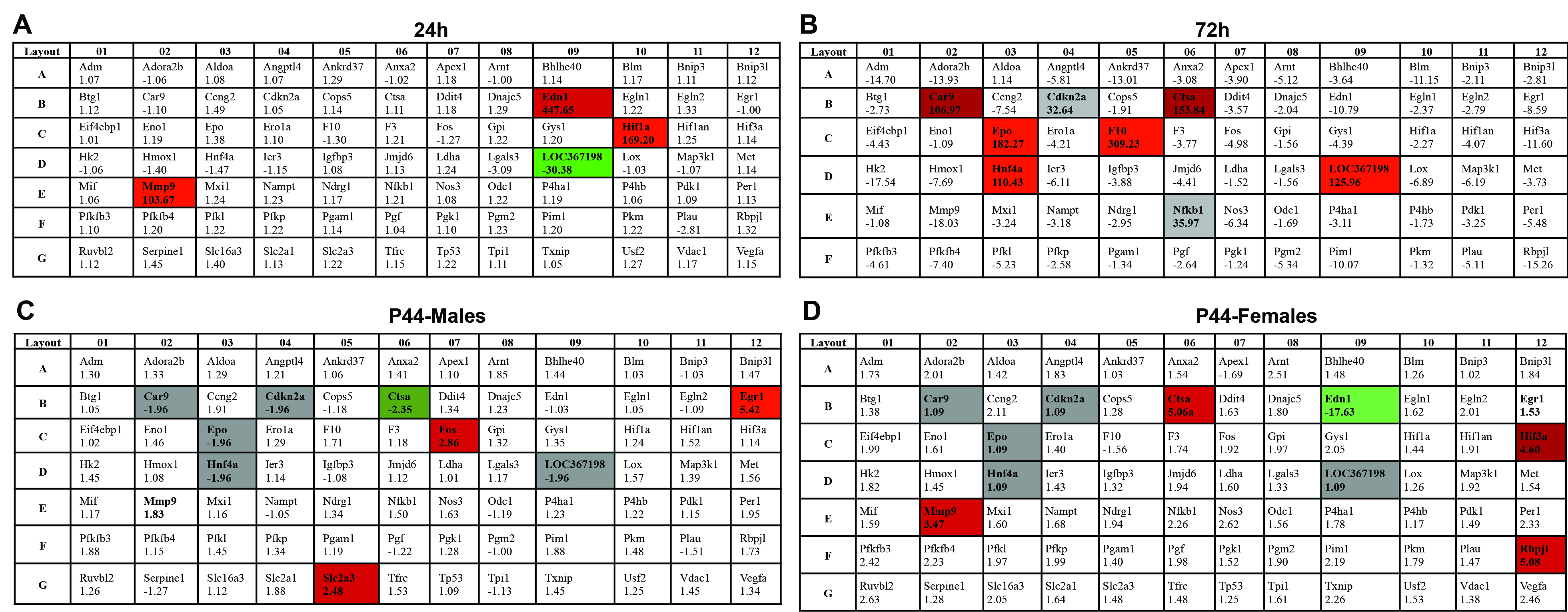

Supplement: Supplementary file 2 — Additional file 2: Figure S2. Log2 fold change of gene expression in the ipsilateral vs contralateral hemisphere of rats induced by Hypoxia ischemia after 24 h (A) 72 h (B) 4 weeks in males (C) and females (D). [file 12974_2019_1595_MOESM2_ESM.tif]

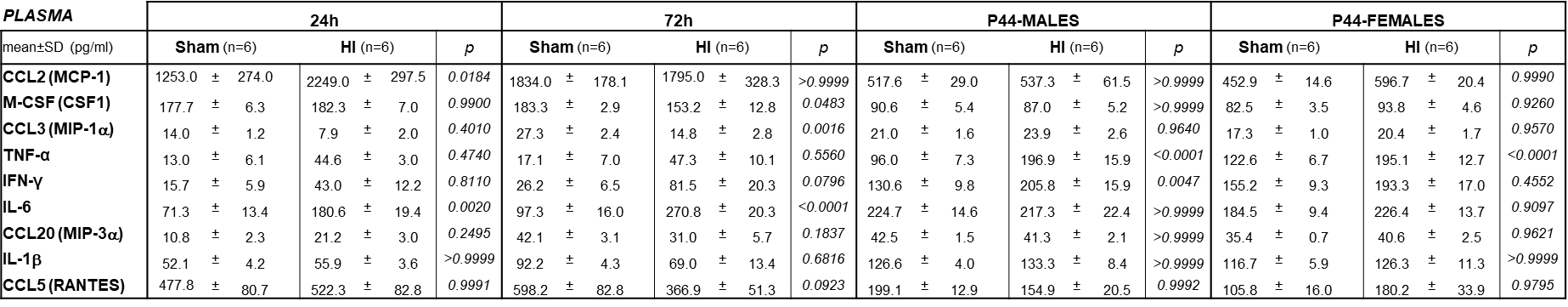

Supplement: Supplementary file 3 — Additional file 3: Table S1. Raw data of plasma level of different cytokines, chemokines and growth factors in sham and HI rats at different time points. Results are presented as mean ± SEM (pg/mL), with the P value (one-way ANOVA and Tukey’s multiple comparison test). [file 12974_2019_1595_MOESM3_ESM.tif]

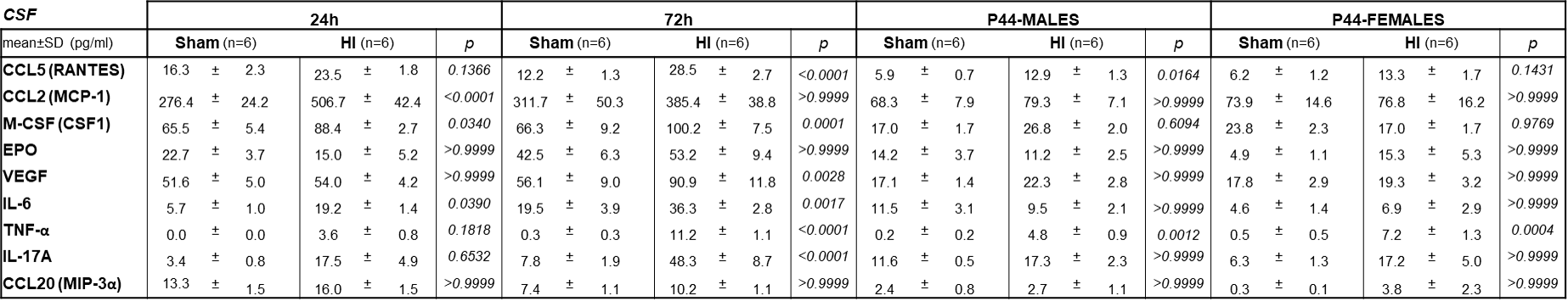

Supplement: Supplementary file 4 — Additional file 4: Table S2. Raw data of CSF level of different cytokines, chemokines and growth factors in sham and HI rats at different time points. Results are presented as mean ± SEM (pg/mL), with the P value (one-way ANOVA and Tukey’s multiple comparison test). [file 12974_2019_1595_MOESM4_ESM.tif]
